# Repeatability and Reproducibility of SMTube Measurement in Dry Eye Disease Patients

**DOI:** 10.1155/2021/1589378

**Published:** 2021-10-08

**Authors:** Yiran Hao, Lei Tian, Kai Cao, Ying Jie

**Affiliations:** ^1^Beijing Institute of Ophthalmology, Beijing Tongren Eye Center, Beijing Tongren Hospital, Capital Medical University and Beijing Ophthalmology and Visual Sciences Key Laboratory, Beijing 100730, China; ^2^Beijing Advanced Innovation Center for Big Data-Based Precision Medicine, Beihang University and CapitalMedical University, Beijing, China

## Abstract

**Purpose:**

To evaluate the intraobserver repeatability and interobserver reproducibility of Strip Meniscometry Tube (SMTube) and determine the correlations among the SMTube measurements and other ocular examinations in dry eye disease (DED) patients.

**Methods:**

The study recruited 73 eyes of 49 DED patients. Every subject was subjected to the following five measurements sequentially: the Ocular Surface Disease Index (OSDI) questionnaire, Tear Meniscus Height (TMH) assessment, SMTube measurements, tear film breakup time (BUT) examination, and Schirmer I test (SIT). The repeatability and reproducibility of the measurements were assessed by the intraclass correlation coefficient (ICC) and the Bland–Altman analysis, and the correlations were evaluated by the Spearman rank-order correlation.

**Results:**

The repeatability and reproducibility of the SMTube measurements were good in DED patients. The ICCs between the repeatability and reproducibility of the SMTube measurements were 0.789 and 0.741, respectively, and the Bland–Altman 95% limits of the repeatability and the reproducibility were −1.726 to 1.658 and −0.967 to 1.474, respectively (all *P* < 0.01). The SMTube measurements had correlations with TMH, BUT, and SIT. The Spearman rank correlation coefficients between SMTube and TMH; SMTube and Schirmer I test; and SMTube and BUT were 0.632, 0.617, and 0.653, respectively (all *P* < 0.01).

**Conclusions:**

The measurements of the SMTube may provide a novel, swift, noninvasive, and convenient approach to screen and diagnose DED with acceptable repeatability and reproducibility and specific correlations with TMH, BUT, and SIT.

## 1. Introduction

Dry eye disease (DED), a type of multifactorial disease accompanied by various ocular symptoms, is mainly caused by the instability of the tear film and the imbalance of the microenvironment of the ocular surface, which could accompany the inflammation and damage of the ocular surface and neurosensory disorders, which is according to the consensus of DED in China (2020). Millions of people worldwide suffer from DED as it is one of the most prevalent ocular disorders; the prevalence of DED in China has been estimated to be over 30% [1]. Ocular pain is usually reported as the major complaint by patients, accompanied by light sensitivity, dryness, irritation, foreign-body sensation, and discomfort, which negatively influences the patients' daily life [2]. Although DED has been considered a common disease, the “gold standard” signs or symptoms still do not exist [3]. Clinically, the Schirmer I test and the tear film breakup time (BUT) assessment has been used widely to diagnose DED, while the Ocular Surface Disease Index (OSDI) questionnaire in the Chinese version has been used to evaluate patients' subjective symptoms [3]. As traditional approaches are invasive and quite time consuming, it is imperative to find a concise and noninvasive testing method for the busy outpatient service.

The tear meniscus is a reservoir containing the tear film [4]; its reduction has been reported as a sign of aqueous-deficient dry eye (ADDE) [3]. Therefore, measurements to assess tear meniscus height (TMH) and tear meniscus area (TMA) are essential for diagnosing DED. The commonly used approaches to examine TMH include slit-lamp examination with fluorescein dye staining, anterior segment optical coherence tomography (OCT), Keratograph 5M (K5M), and strip meniscometry (SM) [4–6]. Dogru et al. reported SM as a novel, simple, swift, and noninvasive method for measuring the tear meniscus volume, which can be widely applied in screening, diagnosis, and therapeutic evaluation of DED [7]. Ibrahim et al. have also confirmed SM's efficiency in DED diagnosis [8]. Strip Meniscometry Tube (SMTube) is produced to perform the strip meniscometry measurement. With a convenient structure and an appropriate standard, SMTube allows medical staff to conduct the test swiftly and accurately. Previous studies have mainly focused on the correlations among SMTube assessment and other ocular examinations, while the repeatability and reproducibility of SMTube have hardly been studied [5, 7, 9–11]. Therefore, this study explored the intraobserver repeatability and interobserver reproducibility of the SMTube assessment as a reliable and guaranteed clinical tool, besides assessing the correlations among the SMTube and the Schirmer I test, BUT, and TMH.

## 2. Methods

### 2.1. Subject Recruitment

The prospective study included 73 eyes of 49 DED patients who visited our outpatient department. The DED diagnosis was confirmed based on the consensus of DED in China (2020): at least 1 of 7 symptoms including dryness, sandiness, burning, tiredness, discomfort, redness, and blurred vision with OSDI questionnaire score ≥13 and (1) fluorescein tear film breakup time (FBUT) ≤5 s or a nonanesthesia Schirmer Ι test value ≤5 mm/5 min; (2) 5 s < FBUT ≤ 10 s or 5 mm/5 min < nonanesthesia Schirmer Ι test ≤10 mm/5 min, accompanied with corneal fluorescein staining score.

Patients over 18 years of age, the same ethnicity (Chinese), and willing to participate in the study were included after signing the informed consent. However, patients with any corneal or ocular surgery, any history of Stevens Johnsons Syndrome or Sjögren syndrome, or other systemic illness or risk factors were excluded.

The data were collected between February and April 2021 in Beijing Tongren Hospital, Beijing, China. All subjects signed an informed consent form in accordance with the tenets of the Declaration of Helsinki. The study was approved by the institutional review board of Beijing Tongren Hospital, Beijing, China.

### 2.2. Ocular Examinations

Each patient was asked to complete ocular examinations in the following order: TMH assessment, SMTube measurements, BUT examination, and the Schirmer I test. At least 5 min of rest was arranged between each SMTube measurement and at least 10 min rest between other examinations to let the function of the ocular surface recover. Meanwhile, all subjects were asked to complete the OSDI questionnaire (scores ranging from 0 to 100) before all ocular examinations.

#### 2.2.1. Dry Eye Symptoms Questionnaire

A validated Chinese version of the OSDI questionnaire was used to assess the perceived symptoms as well as the quality of life of the patients [12]. The OSDI questionnaire consists of 12 questions segmented into 3: ocular symptoms, vision-related function, and environmental triggers [13]. The OSDI questionnaire is scored on a range of 0–100, with a higher score representing severe disability. Depending on the outcome, the patients were divided into four groups: normal (0–12 points), mild DED (13–22 points), moderate DED (23–32 points), and severe DED (33–100 points) [13, 14].

#### 2.2.2. Strip Meniscometry Tube Measurement

SMTube is a thin strip (length: 85 mm, width: 7 mm, and height: 0.3 mm) with a capillary absorber in the center and two columns of scale on both sides to measure the tear meniscus volume. The examiner held the center part of the strip and immersed the tip into the tear meniscus of the lower eyelid for 5 s to absorb tears. The SMTube attached to the tear meniscus absorbed the tears into the ditch and the strip color turned blue, indicating the volume. At the end of 5 s, the strip was taken out and the blue stained column length was measured. The length of the stained column for normal people was equal to or greater than 5 mm, whereas it was less than 5 mm for DED patients [5, 7, 10, 15].

#### 2.2.3. Fluorescein Tear Film Breakup Time

Before the BUT measurement was carried out, an aseptic fluorescein strip moisturized with normal saline was dipped into the patient's conjunctival sac in both eyes. After dipping the fluorescein dye, the subject was asked to blink several times to ensure that the tear film was stained evenly. Then, the subject was asked to keep his eyes open. The examiner observed the tear film through a cobalt-blue filter by using a slit-lamp microscope. The interval between the last complete blink and the appearance of the first corneal black spot in the stained tear film was recorded three times by using a stopwatch, and the mean of the records was calculated as the BUT, which was regarded abnormal if it was less than or equal to 5 s.

#### 2.2.4. Schirmer I Test

The Schirmer I test was performed without anesthesia to assess aqueous tear production. Standardized Schirmer I test strips were placed over the junction of the middle and outer third of the inferior lid, and they were left for 5 min with the eyes closed. The length of the strip that got wet (in millimeters) was read as the outcome, and a reading less than or equal to 5 mm was considered abnormal.

#### 2.2.5. TMH Measurement

The Oculus Keratograph was applied to assess the TMH. The subjects were required to rest their chin on the chin rest with their foreheads pressed against the forehead band and watch the fixation target straight inside the device. The TMH images were captured and measured perpendicular to the lid margin at the central point relative to the pupil center using an integrated ruler.

### 2.3. Repeatability and Reproducibility of the SMTube Measurements

Two individual tests were performed by two different clinicians at 10–15 min intervals in random order to measure the interobserver reproducibility. Two consecutive measurements were carried out at 5–10 min intervals by the same clinician to measure the intraobserver repeatability. All analyzers were masked to hide the subjects' clinical and demographic details. All assessments were conducted in a dimly lit room (temperature 20–25°C and humidity 30–40%) between 8 am and 4 pm in a single day.

### 2.4. Statistical Analysis

As for a study design with 2 repeated measures, the uncertainty was set to be 20% in the repeatability and reproducibility result, which means the sample size for precision studies must be over 48 according to the following formula [16]:

(1) $$\begin{array}{c} 1.96\frac{\text{Sw}}{\sqrt{2n\left(n^{\prime }-1\right)}}=0.2\text{Sw}, \end{array}$$

where Sw: within-subject standard deviation; *n*: number of the subject; and *n'*: number of repeated measurements.

SPSS version 21.0 (SPSS, Inc., Chicago, IL, USA) was used to conduct the statistical analysis. The intraclass correlation coefficient (ICC) was calculated from the two consecutive tests and the two individual tests to assess the intraobserver repeatability and the interexaminer reproducibility, respectively; ICC ≥0.8 indicated good reliability. The Bland–Altman analysis was also used to determine the repeatability and reproducibility. The Spearman rank-order correlation coefficient was calculated to evaluate the correlation among SMTube measurement and other ocular examinations; a coefficient ≥0.7 indicated good reliability. The standard deviation (SD) and coefficient of variance (CV) were determined to assess the fluctuation of certain ocular examination parameters. All *P* values were two sided and were considered statistically significant at *P* < 0.05. The intercorrelation between paired eyes of individuals was eliminated using a generalized linear mixed model.

## 3. Results

### 3.1. Demographics

A total of 73 eyes of 49 patients were recruited for the study, and the confidence in the estimate is 0.16 according to the sample size.  Table 1 displays the mean values and coefficient of variance of the SMTube measurement, TMH assessment, Schirmer I test, and BUT test.

### 3.2. Intraobserver Repeatability and Interobserver Reproducibility

**Figure 1:**
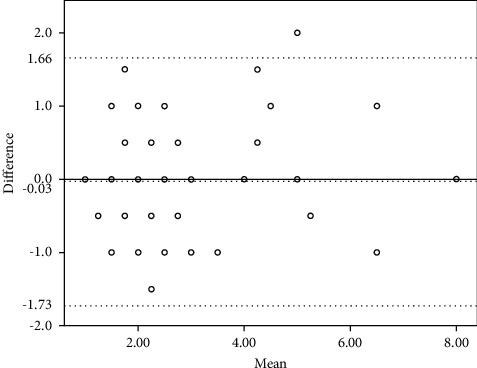
Bland–Altman plots comparing the intraobserver repeatability of the two consecutive repeated tests. The difference of the measurements (difference) is plotted on the vertical axis, and their mean is plotted on the horizontal axis (mean). Despite the zero line, the middle horizontal dotted line represents the mean difference and the two horizontal lines, one above and the other below, are the 95% limits of agreement of the intraobserver repeatability.

**Figure 2:**
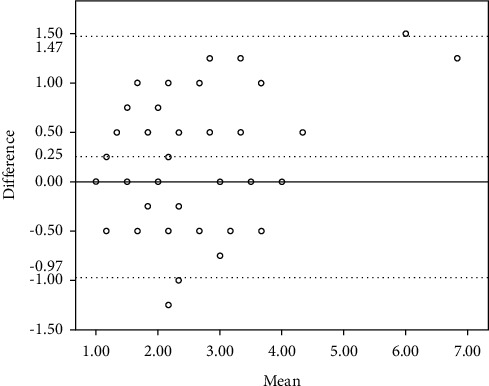
Bland–Altman plots comparing the interobserver reproducibility of the two individual tests. The difference of the measurements (difference) is plotted on the vertical axis, and their mean is plotted on the horizontal axis (mean). Despite the zero line, the middle horizontal dotted line represents the mean difference and the two horizontal lines, one above and the other below, are the 95% limits of agreement of the interobserver reproducibility.

Table 2 shows the ICC, 95% confidence interval for ICC, and Bland–Altman 95% limits of the SMTube measurement for the two consecutive repeated tests completed by the same clinician and the two individual tests performed by different clinicians. The ICC values were 0.789 and 0.741, respectively, both of which were more than 0.7, and the *P* values were less than 0.01, thus indicating satisfying reliability. The Bland–Altman 95% limits of the intraobserver repeatability and the interobserver reproducibility were −1.726 to 1.658 and −0.967 to 1.474, respectively, which are displayed graphically in Figures 1 and 2. The mean difference of intraobserver repeatability does not have a significant difference from zero line by using the one-sample *t*-test, with *P* > 0.05. However, the mean difference of interobserver reproducibility has a significant difference from zero line by using the one-sample *t*-test, with *P* < 0.05. Therefore, the intraobserver repeatability and the interobserver reproducibility of the SMTube measurement were good.

### 3.3. Correlations among SMTube Measurements and DED Parameters

**Figure 3:**
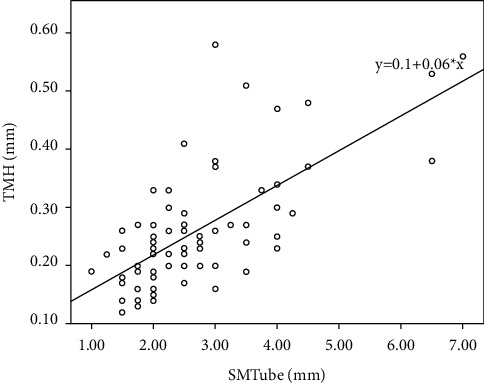
The Spearman correlation by the rank test between the SMTube test and TMH measurement (*P* < 0.01, *ρ* = 0.632).

**Figure 4:**
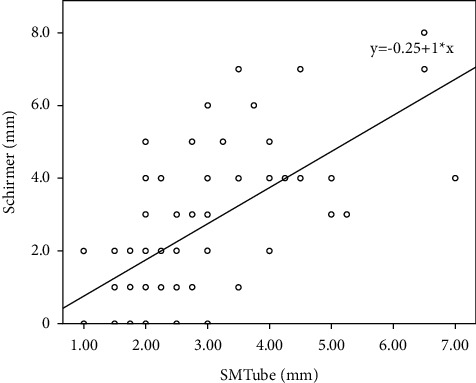
The Spearman correlation by the rank test between the SMTube test and Schirmer I test (*P* < 0.01, *ρ* = 0.617).

**Figure 5:**
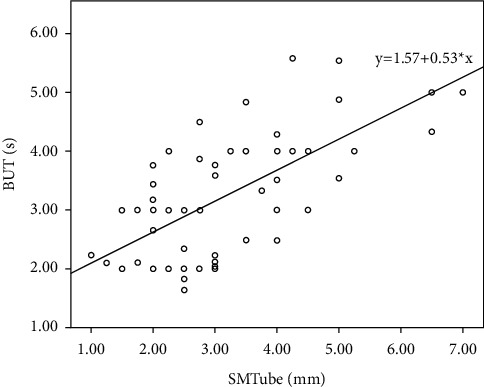
The Spearman correlation by the rank test between the SMTube test and BUT test (*P* < 0.01, *ρ* = 0.653).

Table 3 and Figures 3–5 show the mean values and the Spearman rank correlation coefficients among SMTube measurements and other ocular examination parameters. The Spearman rank correlation coefficients were more than 0.6 for SMTube and TMH (0.632), SMTube and Schirmer I test (0.617), and SMTube and BUT (0.653), indicating positive correlations, while the *P* values were less than 0.01. Thus, SMTube measurement and other DED parameters had positive correlations.

## 4. Discussion

In 2020, the consensus of DED in China refined DED as “a multifactorial chronic disease of the ocular surface characterized by a loss of homeostasis of the tear film or imbalance of the ocular surface microenvironment caused by abnormal quality, quantity, and dynamics of tears, which can be accompanied by ocular surface inflammation and damage and neurosensory abnormalities, resulting in a variety of ocular discomfort symptoms and/or visual dysfunction.” However, the diagnosis of DED remained complicated due to a lack of consistency in the subjective symptoms, clinical signs, ocular test results, and variation in personal cognition of ocular sensation and pain threshold [17]. Diversified approaches can be applied for the diagnosis, classification, evaluation, and supervision of dry eye, including corneal and conjunctival vital dye staining, meibomian-gland grading, the Schirmer I test (with or without anesthesia), questionnaires, tear film stability (tear film breakup time), tear osmolarity, tear film interferometry, and InflammaDry immunoassay [2]. However, due to the limitation of time, space, and outpatient service expenditure, many diagnostic tests are not available for specialty settings, and only some convenient tools are widely used, including the Schirmer I test, BUT assessment, dry eye symptoms questionnaires, and other ocular examinations with special equipment.

Previous studies have shown that the repeatability of some procedures clinically used to diagnose and monitor dry eye syndromes, such as the Schirmer I tests, BUT assessment, and presence or absence of inferior corneal fluorescein staining, still needs to be improved [18, 19]. Some approaches such as the Schirmer tests take a long time, while others such as the TMH assessment require special facilities. Therefore, finding a novel, swift, convenient, and reliable tool to diagnose and evaluate DED is necessary.

Strip meniscometry is a relatively novel, rapid, noninvasive, and convenient approach to assess the tear meniscus volume. It takes only 5 s to carry out the whole process with minimal invasion. Additionally, its conciseness and easy application are a great help to clinical staff. Previous studies have observed a significant difference in the SMTube measurements between DED patients and normal people. Good sensitivity and specificity of SMTube measurement results indicated it to be a feasible way to diagnose and evaluate DED [5, 8, 10, 20, 21].

Repeatability, calculated by the ICC, evaluates the accuracy of measurements or clinical trials by showing the proportion of variation that can reappear in the repeated tests of the same subjects or groups by the same examiner, indicating that it could become a comparable parameter across studies [22]. Reproducibility refers to expecting the same results when a second researcher uses the same raw data or materials to implement the same procedure or statistical analysis [23]. Repeatability and reproducibility are the necessary conditions for any new instrument or method in clinical diagnosis and treatment.

The current study's statistical results confirm good repeatability and reproducibility of the SMTube assessment according to the outcome of ICCs and the results from Bland–Altman plots. Although the mean difference of reproducibility has a significant difference with zero line, the Bland–Altman plots still show relatively good reproducibility with only 3 of 35 out of the 95% limits of agreement, and the ICC is also over 0.7. The results suggest that the ocular examination could be a novel, reliable way to screen DED patients. Most previous studies illustrate that the ocular examinations used in the study including noninvasive BUT, TMH, and the Schirmer test have confirmed repeatability and reproducibility [6, 24–26], despite some findings highlighting the need for improvement in the repeatability of the Schirmer tests and TMH among DED patients [18, 26]. The statistical outcome of SMTube measurements in the current study confirmed satisfactory results for repeatability and reproducibility, suggesting that it was reliable to be utilized in screening and diagnosing DED.

We also tested the correlations among SMTube assessment and other frequently used ocular measurements, including the Schirmer test, BUT, and TMH. Although the outcome did not show high correlations, several previous studies have proved that SM had a high correlation with the Schirmer I test [7, 9], BUT [7, 11], and TMH [5, 10]. As discussed before, SM uses SMTube to measure the volume of tear reserve in the tear meniscus. The tear meniscus acted as a reservoir for the strip in the Schirmer I test. Dogru et al. [7] and Miyasaka et al. [9] demonstrated that the SM results correlated with those of the Schirmer test because both tests relied on the tear meniscus volume. The BUT measurement evaluates the stability of a tear film. Patients with ADDE have diminishing tear secretion volume accompanied by an unstable tear film and decreasing tear meniscus volume. Dogru et al. [7] and Shinzawa et al. [11] reported a good correlation between BUT and SMTube measurements. The TMH correlated with the tear meniscus volume and, hence, correlated with the SM results. Ibrahim et al. [8], Lee et al. [5], and Shinzawa et al. [10] illustrated a high correlation of the TMH assessment with the SMTube test. Therefore, SM through SMTube was a feasible and reliable method to screen, evaluate, and diagnose DED due to reliable intraobserver repeatability, interobserver reproducibility, and satisfied correlations with other common ocular examinations. Moreover, it provides clinical staff a swift and convenient inspection method during the busy outpatient service. The results of this study did not relate well with previous studies due to a lack of measurement of healthy eyes. As discussed previously, the repeatability of common ocular examinations in the DED group is a bit lower than that in the healthy group, indicating that the correlation might also be influenced.

A possible limitation or bias of the present study was the limited number of subjects that might not be enough to represent the results of all DED patients. We did not have a control group and, therefore, cannot display the difference between the DED group and the control group.

## 5. Conclusions

Strip meniscometry tube is a novel, swift, noninvasive, and convenient approach to screen, diagnose, and evaluate DED by assessing the tear meniscus volume. Our study observed reliable intraobserver repeatability and interobserver reproducibility in SMTube measurements and relatively satisfied correlations with commonly used ocular examinations, including the Schirmer I test, BUT, and TMH, which is consistent with previous study results. Strip meniscometry tube will be of great assistance for clinical staff in enhancing the efficiency of the diagnosis and treatment in hectic outpatient service.

## Figures and Tables

**Table 1:** Mean values and coefficient of variance of the SMTube measurement, TMH assessment, Schirmer I test, and BUT test.

Parameters	*n*	Mean ± SD^a^	CV^b^ (%)
SMTube first (mm)	73	2.596 ± 1.490	57.39
SMTube average (mm)	73	2.613 ± 1.361	52.11
TMH (mm)	73	0.261 ± 0.103	39.70
Schirmer I (mm)	73	2.493 ± 1.959	78.57
BUT (s)	73	3.126 ± 0.982	31.42

^a^SD: standard deviation; ^b^CV: coefficient of variance.

**Table 2:** Intraobserver repeatability and interobserver reproducibility of SMTube measurement.

Parameters	*n*	ICC^a^	95% CI^b^ for ICC	*P* value	Bland–Altman 95% limits of agreement
*Intraobserver repeatability*					
SMTube (mm)	73	0.789	0.700 to 0.854	<0.001	−1.726 to 1.658
*Interobserver reproducibility*					
SMTube (mm)	73	0.741	0.612 to 0.827	<0.001	−0.967 to 1.474

^a^ICC: intraclass correlation coefficient; 95% CI^b^: 95% confidence interval for the mean.

**Table 3:** Correlations among SMTube measurement and the DED^a^ parameters.

Parameters	*n*	Mean ± SD^b^	Spearman's rho	*P* value
TMH^c^ (mm)	73	0.261 ± 0.103	0.632	<0.001
Schirmer I (mm)	73	2.493 ± 1.959	0.617	<0.001
BUT^d^ (s)	73	3.126 ± 0.982	0.653	<0.001

^a^DED: dry eye disease; ^b^SD: standard deviation; ^c^TMH: tear meniscus height; ^d^BUT: tear breakup time.

## Data Availability

The data used to support the findings of this study are available from the corresponding author upon request.
